# Prevalence and Perinatal Outcomes of Singleton Term Breech Delivery in Wolisso Hospital, Oromia Region, Southern Ethiopia: A Cross-Sectional Study

**DOI:** 10.1155/2017/9413717

**Published:** 2017-11-23

**Authors:** Temesgen Debero Mere, Tilahun Beyene Handiso, Abera Beyamo Mekiso, Markos Selamu Jifar, Shabeza Aliye Ibrahim, Degefe Tadele Bilato

**Affiliations:** ^1^Homecho Hospital, SNNPRS, Homecho, Ethiopia; ^2^Institute of Health, Jimma University, Department of Epidemiology, Jimma, Ethiopia; ^3^Department of Public Health, Wolaita Sodo University, Sodo, Ethiopia; ^4^Misha District Health Office, SNNPRS, Morsito, Ethiopia; ^5^Department of Statistics, Wachemo University, Hossana, Ethiopia

## Abstract

**Background:**

Breech deliveries have always been topical issues in obstetrics. Neonates undergoing term breech deliveries have long-term morbidity up to the school age irrespective of mode of delivery.

**Objective:**

To determine prevalence and perinatal outcomes of singleton term breech delivery.

**Methods:**

Hospital based cross-sectional study was conducted on 384 participants retrospectively. Descriptive and analytical statistics was used.

**Result:**

A total of 384 breech deliveries were included. Prevalence of singleton breech deliveries in the hospital was 3.4%. The perinatal outcome of breech deliveries was 322 (83.9%). Adverse perinatal outcome of singleton term breech delivery was significantly associated with women's age of greater than or equal to 35 years (AOR = 2.62, 95% CI = 1.14–6.03), fully dilated cervix (AOR = 0.48, 95% CI = 0.25–0.91), ruptured membrane (AOR = 5.11, 95% CI = 2.25–11.6), and fetal weight of <2500 g (AOR = 6.77, 95% CI = 3.22–14.25).

**Conclusion:**

Entrapment of head, birth asphyxia, and cord prolapse were the most common causes of perinatal mortality. Factors like fetal weight <2500 gm, mothers of age 35 years and above, those mothers not having a fully dilated cervix, and mothers with ruptured membrane were associated with increased perinatal mortality.

## 1. Background

Breech presentation is a longitudinal lie of the fetus with the caudal pole (buttock or lower extremity) occupying the lower part of the uterus and cephalic pole in the uterine fundus [1]. There are three types of breech presentation. In the frank breech position (48 to 73%), both hips are flexed and both knees are extended. In the complete breech position (4.6 to 11.5%), both hips and both knees are flexed. In the incomplete breech position (12.4 to 40.5), one or both hips are not completely flexed [2].

The breech fetus is at increased risk of harm during delivery because cord compression between the cervix and body must occur as the breech crowns and because the after-coming shoulders, head, and arms are at greater risk of harm from dystocia.

Breech deliveries have always been topical issues in obstetrics because of the very high perinatal mortality and morbidity. These are due to combination of trauma, birth asphyxia, prematurity, and malformation. In addition 19.4% of neonates undergoing term breech deliveries have long-term morbidity up to the school age irrespective of mode of delivery [3].

The predisposing factors for breech deliveries include maternal factors (foetopelvic disproportion, soft tissue dystocia, uterine anomalies; pelvic tumors (myoma, ovarian neoplasm, etc.), and grand multipara); placenta factors (placenta previa, cornual placenta (it contributes 73 percent)); liquid factors (polyhydramnios, oligohydramnios); and cord factors (very long cord and very short cord). Fetal factors include multiple pregnancies and congenital anomalies (fetal anomalies have been observed in 18 percent of preterm breech and 4–8 percent of term breech deliveries). Other purported risk factors include primiparity, female gender, maternal anticonvulsant therapy, older maternal age, fetal growth restriction, and previous breech presentation [4, 5].

The wide ranges of management policies have been instituted with the aim of reducing perinatal morbidity and mortality. External cephalic version (ECV) is one of such policies. A successful ECV leads to a more favourable presentation and reduces the incidence of breech deliveries, perinatal morbidity, and mortality. This was the reason the Royal College of Obstetricians and Gynaecologists in 2001 recommended that all women with an uncomplicated breech presentation at term be offered an ECV [6, 7]. The safest route of delivery for breech presentations has long been a topic of debate. The Term Breech Trial study (TBT) revealed that perinatal mortality or serious neonatal morbidity was significantly lower for the planned caesarean section group than for the planned vaginal birth group [7].

Breech presentation is the most common form of malpresentation, accounting for 3%-4% of all deliveries at term. Persistent breech presentation may be associated with abnormalities of the baby, the amniotic fluid volume, the placental localization, or the uterus. It may be due to an otherwise insignificant factor such as cornual placental position or it may apparently be due to chance [8, 9].

Morbidity and mortality for breech infants and mothers is most related to inclusion and exclusion criteria adhered to by the hospital for determining mode of delivery, the competence of the attending physician, and the expectation of the mother rather than the mode of delivery. In general countries that perform more vaginal breech births have birth outcomes that are as good as or better than caesarean section outcomes. Countries that perform few vaginal breech births have birth outcomes that are worse than those for caesarean section births. In many countries breech vaginal birth has higher morbidity and mortality risks for babies, but the risk is still relatively low [10–12].

From a study done in Scotland on the breech deliveries, there were 11 (28.0 per 10,000 births) delivery related perinatal and neonatal deaths in women who had a vaginal breech delivery. The risk of death (24.3 per 10,000 births) was similar among women having a prelabour caesarean delivery, but only approximately half of these losses were due to asphyxia. There were four (8.1 per 10,000 births) perinatal and neonatal deaths in those who had a postlabour emergency caesarean delivery and two of these were due to asphyxia. There were seven (3.5 per 10,000 births) perinatal and neonatal deaths in women who had a planned caesarean delivery. The absolute risk of asphyxia perinatal death was 25.5 per 10,000 deliveries for women who had a vaginal breech delivery, 12.2 per 10,000 deliveries for women who had a prelabour emergency caesarean delivery, and 4.1 per 10,000 deliveries for women who had a postlabour emergency caesarean delivery [13]. The combined stillbirth and neonatal mortality rate was 3.9 per thousand births (22 fetal deaths and 10 neonatal deaths). Seven fetal deaths occurred at or after 39 weeks. Fetal or neonatal death or serious neonatal morbidity without lethal congenital anomalies was reported for 129 infants, or 1.59% of the entire sample. A 5-minute APGAR score less than 5 was significantly more frequent in the planned vaginal group as much as 4 [14].

The total mortality rate in the breech presentation group was 0.92% (*n* = 60). The rate of intrapartum and early neonatal death among infants born vaginally was 212248 (0.09%). The numbers and incidences of low APGAR scores in the vaginal group was as high as 69 (3.1%) [15]. The perinatal mortality (PNM) of breech infants was 79/1000. PNM due to CS in breech is 6/1000 and in breech vaginal delivery is 72/1000 [16].

The overall perinatal mortality was significantly (*P* = 0.011) high among breech babies, as much as 7.5%. There was more stillbirths and early neonatal deaths in breech deliveries (3.75%); LBW was significantly (*P* = 0.001) high in breech deliveries (0.16%). Breech babies had significantly lower APGAR scores (*P* = 0.026) at five minutes. Sixteen percent of breeches were admitted to the NICU [17]. The perinatal mortality rate was 32 per 1000 births. The main causes of death were head entrapment (0.8%), cord prolapse (0.8%), and severe asphyxia (0.8%). The overall incidence of neonatal traumatic morbidity was 2.6% and the incidence of maternal morbidity was 23.3 [18].

In one study done in Yekatit 12 Hospital, Addis Ababa, Ethiopia, there was higher perinatal mortality compared to the total number of deliveries; fetuses with low birth weight show a higher mortality rate. There is also a twofold increase in perinatal death in patients without antenatal care [19].

A study done in Hawassa and Jimma shows that the overall perinatal mortality (intrapartum and early neonatal deaths) in the planned vaginal delivery in term singleton breech was as high as 253 (0.3%). Thus, the risk of perinatal mortality was high in planned vaginal deliveries, 1 in 300 deliveries. The overall rates of birth trauma in the planned vaginal deliveries was as high as 1 in 150 (0.7%); the absolute risk of APGAR score <7 was high in the planned vaginal deliveries groups (2.4%). The absolute risk of neonatal asphyxia in the planned vaginal deliveries was as high as 3.3% [10].

Factors associated with increased risk of breech delivery in Flanders (Northern Belgium) are low gestational age and low birth weight, increasing maternal age, primiparity, history of caesarean section, female gender, and presence of congenital malformation [20]. Breech delivery is associated with high perinatal morbidity and mortality, if there is low birth weight and prematurity [21].

It is important to know prevalence and fetal outcome in singleton breech presentation in reducing perinatal morbidity and mortality in our country and the absence of complete data on the prevalence and fetal outcome in singleton breech presentation at hospital level in our country and geographical variation of factors associated with it, except few studies, in north and central Ethiopia [10, 19].

The prevalence of singleton term breech deliveries in most of studies was almost similar to what was stated by world health organization which is affected by factors like sociodemographic related factors, maternal and obstetric related factors, and child related factors. In our nation, Ethiopia, early diagnosis and intervention are not equally performed at all setups due to lack of human resources and diagnostic facilities, inadequate transportation facilities, poor ANC visit which might contribute to difficulty of management and increased risk of complications and poor outcomes. It is important to know prevalence in breech presentation and perinatal outcome in our setups. Knowing the prevalence in breech presentation and perinatal outcomes will help as know the most frequent complications in this setup, which in turn helps to develop guidelines on breech presentation and prevention of complications. Therefore, this study fills the existing information gap and will improve existing knowledge.

## 2. Methods and Participants

### 2.1. Study Area and Period

The aim of this study is to assess prevalence of breach presentation and perinatal outcomes of term singleton breech delivery and its associated factors at Wolisso Hospital, Oromia Region, Southern Ethiopia, in 2016.

The study was conducted from January 1, 2016, to January 30, 2016, on breech delivery of 3 years retrospective data from January 1, 2013, to December 31, 2015, at Wolisso Hospital, Woliso town, which is the Capital Town of Southwest Shewa Zone in Oromia Region, located about 116 km from Addis Ababa in the south west. The zone is one of the eighteen zones of Oromia Region and has eleven districts inhabited by a total of 1,173,363 populations. St. Luke Hospital is one of the two hospitals in the zone and gives clinical services to the population of the zone and nearby regions. It was established and began giving service since January 1, 2001. It is owned by Catholic Church and staffed with expatriates especially by doctors with African, CUUAM, and Italian nongovernmental organization (NGO). It provides outpatient and inpatient service and the average number of daily patients in the hospital is 304 in 2011. It has a total of 200 beds in different wards, two operation theatre complexes, and outpatient departments for all major medical services in addition to psychiatry, ophthalmology, and dentistry, VCT, PMTCT, and ART. There are also Physiotherapy and therapeutic feeding centers and mothers waiting area for pregnant women at risk. In addition, the hospital also provides public health services both at static and outreach sites.

### 2.2. Study Design

Descriptive and analytic hospital based cross-sectional study was conducted on prevalence and perinatal outcomes of singleton term breech delivery in the past three years at St. Luke Catholic Hospital Southwest Shewa Zone in Oromia Region.

Cards of women who gave birth of breech delivery from January 1, 2013, to December 31, 2015, were obtained.

#### 2.2.1. Inclusion Criteria

Mothers with singleton breech presentation who had either vaginal, C/S, or instrumental birth in St. Luke Catholic Hospital from January 1, 2013, to December 31, 2015, were included.

#### 2.2.2. Exclusion Criteria

Mothers whose charts were lost.

- Mothers with preterm breech deliveries.
- Congenital malformed breech deliveries, because congenital malformed fetus can have reduced survival so that can affect outcome of the fetus.

### 2.3. Sample Size Determination and Sampling Technique

#### 2.3.1. Sample Size Determination

The sample size was calculated by the single population proportion formulae considering the following assumptions:

 
*P* = 0.5, because there is no study done in Ethiopia on prevalence of singleton breech
 
*q* or (1 − *p*) = proportion of deliveries without breech deliveries
 
*Zα*/2 = statistic for the level of confidence at 95%, which is 1.96, with 4% margin of error (*e*). The following formula was used for calculating the sample size: *n* = ((*Zα*/2)2 × *p*(1 − *p*))/*d*2
 
*n* = (1.96)^2^ × 0.5 × 0.5/(0.05)^2^ = 384.

#### 2.3.2. Sampling Technique

First, obstetrics and operative records from obstetric ward and major operation registry book in the operation room were reviewed to identify women who presented with breech presentation and delivered from January 1, 2013 to December 31, 2015. Next, using card number of clients, cards were collected from the card room using simple random sampling method. Finally, based on the inclusion and exclusion criteria of the study, cards were selected.

### 2.4. Study Variables

#### 2.4.1. Dependent Variables

- Perinatal outcomes on singleton breech deliveries (1: died, 0: alive)
- Prevalence of singleton term breech deliveries.

#### 2.4.2. Independent Variables

Sociodemographic related factors include age and address. Maternal and obstetric related factors include gestational age, parity, mode of delivery, cervical dilatation, status of membrane, and antenatal care utilization and child related factors include child weight and IUFD and APGAR score.

### 2.5. Data Collection Tool and Procedure

Data collection instrument was developed based on patient cards and charts in English. Data was collected from patient record cards, registration books, and anaesthesia charts available in the hospital by checklist questionnaires using trained data collectors. First card number of patients in the study period was identified from registration books (logbooks), and then their chart was retrieved from card office. Data collectors and supervisor were recruited from the health facilities of the study area. The training was given for about five clinical nurses for data collection and one health officer for supervision for two days on data quality, data collection procedure, ethical issues, and confidentiality of information. Pretest was done on 5%. The principal investigators were strictly monitoring data collectors daily to assure the completeness of filled formats. Finally documents from patient cards were entered in to a structured format by five trained nurses.

### 2.6. Data Processing and Analysis

Data was cleaned and coded with EpiData version 3.2 before being entered into SPSS version 20.0 for analysis. The data was analyzed by SPSS version 20. Frequencies and percentages were used. Chi-square test was performed for categorical variables to check adequacy of cells before performing logistic regression. Bivariate binary logistic regression was used primarily to check crude association of independent variables with prenatal outcome of breech deliveries; then variables found to have *p* value < 0.25 were entered into multivariable binary logistic regression using backward Linear regression method for controlling the possible effect of confounding among independent variables. Before running multivariable binary logistic regression, multicollinearity between the independent variables was checked but not detected. Adjusted odds ratio and corresponding 95% confidence intervals were used to quantify the degrees of association between dependent and independent variables. Results with *p* value < 0.05 were considered as predictors of perinatal outcome. The model fitness for the variables was assessed by Hosmer-Lemeshow goodness of fit test.

### 2.7. Operational Definitions and Definition of Terms

*(1) Fetal Outcome*. Neonatal condition in first 5 minutes after delivery of breech presentation was either alive (healthy or alive asphyxiated or alive with birth injury) or dead.

*(2) Asphyxia*. Asphyxia is the medical condition resulting from deprivation of oxygen to a newborn that lasts long enough during birth process of first 5 minutes that is described in terms of APGAR score.

- Asphyxiated for APGAR <7
- Not asphyxiated for APGAR 7 and above.

*(3) Breech Presentation*. Breech presentation is a longitudinal lie of the fetus with the caudal pole (buttock or lower extremity) occupying the lower part of the uterus and cephalic pole in the uterus. 

*(a) Gestational Age*. Gestational age is the age of the fetus counting from the time of fertilization. 

*(b) Parity*. Parity is the number of live born children a woman has delivered.

- Primipara: those who gave birth only once
- Multipara: those who gave birth above one time
- Grand-multi: those who gave birth above five times.

## 3. Result

During the study period there were 10214 deliveries in Wolisso Hospital in three years. From these deliveries 384 cards were included in the study. Most of the study participants, 141 (36.7%), were within the age group of 25–29 years. Among study subjects 334 (87.0%) were rural, 381 (99.2%) were married, only 3 (0.8%) were single, 382 (99.5%) were attended ANC, 145 (37.8%) had ANC follow-up less than 4 times, 307 (79.9%) did not know their LNMP, 54 (73%) were with 37–42 weeks of GA from LNMP, 266 (69.3%) were with non-fully dilated cervix, 229 (59.6%) were with ruptured membrane, 184 (79.5%) were with rupture of membrane < 8 hours, 281 (73.2%) had assisted vaginal breech delivery, 13 (19.1%) were having indication for CS by footling breech presentation, 362 (94.3%) were with alive intrauterine fetal condition prior to admission, and 322 (83.9%) were alive immediately after delivery.

### 3.1. Obstetrics Condition

Among mothers with term breech presentations, 317 (82.6%) of them gave birth vaginally while 67 (17.4%) of mothers gave birth through caesarean delivery. Among mothers who gave birth vaginally, 281 (73.2%) gave birth through assisted breech delivery, 31 (8.1%) through spontaneous breech delivery, 4 (1%) gave birth through destructive deliveries, and 1 (0.3%) through forceps deliveries.

The common reasons caesarean section is indicated for mothers who gave birth in this study were footling breech, 13 (19.1%), failure to progress, 11 (16.2%), previous c/s scar, 11 (16.2%), fetal distress, 9 (13.2%), contracted pelvis, 4 (5.9%), cord prolapse, 4 (5.9%), PROM, 9 (13.2%), and others, 1 (1.5%).

### 3.2. Perinatal Condition/Related Factors

Of the total singleton 384 breech presentations, 22 (5.7%) were IUFD and 362 (94.3%) were alive prior to admission. Regarding neonatal birth weight, 53 (13.8%) neonates were less than 2500 gm, 262 (68.2%) were 2500–3500 gm, and 69 (18%) was greater than 3500 gm.

### 3.3. Perinatal Outcome

From the total 384 singleton breech presentations, 62 (16.1%) with (95% CI: 13, 20.3) deliveries had bad outcome (death) within the first 5 minutes. The possible cause of bad outcome (death) in 14 (25.5%) was after-coming head entrapment, in 17 (30.9%) was cord prolapse, in 14 (25.5%) was asphyxia, and in 10 (18.2%) was other, whereas among 322 alive babies within 5 minutes, 253 (79.1%) were healthy looking, 22 (6.9%) were with birth injury, 20 (6.2%) were asphyxiated, and 25 (7.8%) were transferred to NICU. This increased poor fetal outcome and fetal complication could be due to only 31 (8.1%) of mothers giving birth through spontaneous breech delivery and 67 (17.4%) of mothers giving birth through caesarean section delivery. Therefore majority of mothers gave birth through assisted breech delivery (281 (73.2%) and 4 (1%) gave birth through destructive deliveries and 1 (0.3%) through forceps deliveries). This condition could increase the chance of fetal distress and can result in poor fetal outcome and increase fetal complication (see Table 1).

### 3.4. Factors Associated with Perinatal Outcome of Breech Delivery

Mothers of age 35 years and above were 2.62 times more likely to have bad fetal outcome within 5 minutes in singleton breech delivery than mothers within 25–29 years age groups. Those mothers with non-fully dilated cervix were 52% less likely to have bad fetal outcome within 5 minutes in singleton breech delivery when compared with mothers with fully dilated cervix. Mothers with ruptured membrane were nearly five times more likely to have bad fetal outcome within 5 minutes in singleton breech delivery than mothers with intact membrane. Neonates with <2500 gm born from singleton breech presentation were 6.77 times more likely to have bad fetal outcome within 5 minutes when compared with neonatal birth weight of 2500–3500 gm (see Table 2).

### 3.5. Prevalence of Perinatal Outcome

This study assessed prevalence and perinatal bad outcome (death) of singleton breech delivery. This study showed that the prevalence of singleton breech delivery was 3.8%; overall it is comparable with worldwide incidence of 3-4%. This study found that the prevalence of fetal bad outcome (death) in singleton breech delivery was 16.1% with 95% CI: 13, 20.3. This study is in line with study conducted in University Teaching Hospital in Eastern Nigeria whose prevalence was 18.9% [22].

### 3.6. Perinatal Outcomes

In this study the perinatal mortality rate is 161 per 1000 term breech presentations. It is higher than study done in ACOG committee; perinatal mortality rate for breech delivery was 66 per 1000 deliveries [23], 3.5 per 1000 deliveries in the study done in Netherlands [24], 9.2 per 1000 deliveries in the study done in Sweden [25], and 3.9 per 1000 deliveries in the study done in France and Belgium [14], but it is lower than the study done in eastern Nigeria (a total perinatal death of 250 per 1000 deliveries) and the study done in Yekatit 12 Hospital, Ethiopia (perinatal mortality rate for breech delivery was 330 per 1,000 deliveries) [19]. Cord prolapse is the leading possible cause of perinatal death. This contradicts with study conducted in Nigeria [26] where the leading cause of death was after-coming head and the research done by Yaoundé Cameroon where the leading cause of perinatal death was related to birth injury [9]. This possible cause of fetal loss maybe related to delay to reach the hospital; this is due to a large proportion of participants being away from Woliso town. These cases of perinatal deaths could be the illustration of the absence of a well-defined selection criteria to allow for trial of vaginal delivery in our setting and early assistance of vaginal breech delivery that means assistance of breech delivery without waiting for spontaneous vaginal delivery up to umbilicus. This increased poor fetal outcome and fetal complication could be due to only 31 (8.1%) of mothers giving birth through spontaneous breech delivery and 67 (17.4%) of mothers giving birth through caesarean section delivery. Therefore, majority of mothers gave birth through assisted breech delivery (281 (73.2%) and 4 (1%) gave birth through destructive deliveries and 1 (0.3%) through forceps deliveries). This condition could increase the chance of fetal distress and can result in poor fetal outcome and increase fetal complication. In addition to what is mentioned above, the reason for poor fetal outcome and increased fetal complication is that 334 (87.0%) mothers were from rural area. In our setting health professionals in rural areas do not manage breech presentation delivery rather than referring to hospitals; this increases delays.

### 3.7. Sociodemographic Factors

This study revealed that advanced age (≥35 years) of mothers in singleton breech delivery has positive association with fetal death until five minutes of delivery. This finding was in agreement with the study findings of the study conducted in London [20] and Belgium [27, 28]. This association could be due to postpartum haemorrhage, macrosomia, uterine atony, and relaxing of abdominal and uterine muscle.

### 3.8. Obstetric Related Factors

In this study those mothers with non-fully dilated cervix have slight protective effect on adverse perinatal outcomes as compared to fully dilated cervix. This finding was similar to the study finding conducted in Siriraj Hospital [29]. This association might be due to the absence of the use of strict selection criteria in selecting cases of trial of vaginal delivery in our hospital and probably to some unskilled health attendants for vaginal breech delivery, prolonged labour, after-coming head arrest, and most of delivering women coming from outside Woliso town and in advanced labour, so they did not have time to investigate with ultrasound to rule out hyperextension of neck and fetal weight. In this study mothers with ruptured membrane were significantly associated with adverse birth outcomes. In line with this, the study conducted in Siriraj Hospital [29] showed significant association with adverse birth outcomes. This association could be due to cord prolapse and infection.

### 3.9. Child Related Factors

In this study neonates with <2500 gm born from singleton breech presentation were significantly associated with adverse birth outcomes, consistent with this study conducted in Belgium [27, 28], Siriraj Hospital [29], and Addis Ababa, Ethiopia [19]. This association could be due to the circumference of the head of the neonate with weight of <2500 g. In contrast, in the study conducted in Basra [30], birth weight of 2500–3500 g was significantly associated with adverse birth outcome as compared with weight of 3500–4000 g and in study conducted in Nigeria birth weight of >3500 g was significantly associated with adverse birth outcome as compared with weight of 2500 g–3500 g.

The limitation of the study is that since the study was facility based review, drawing inferences to the wider community can be difficult. The study did not show long-term complications.

## 4. Conclusion

In this study entrapment of head, birth asphyxia, and prolapsed cord were the most common causes of perinatal mortality. Factors such as fetal weight <2500 gm, mothers of age 35 years and above, those mothers being with non-fully dilated cervix, and mothers with ruptured membrane were significantly associated with increased perinatal mortality.

*Based on the findings of this study the following recommendations are forwarded:*

Perinatal and intrapartum monitoring and evaluation for fetal presentation, weight, wellbeing, and other parameters must be performed.

Obstetricians, midwives, and other health care personnel conducting deliveries should receive a continuous medical education to be updated on how to conduct vaginal breech deliveries and how to resuscitate asphyxiated newborn.

Professionals should manage breech presentation as soon as possible when labour starts to reduce delays to improve fetal outcome.

## Figures and Tables

**Table 1:** Bivariate logistic regression analysis of associated factors with perinatal outcomes among breech deliveries of Wolisso Hospital (*N* = 384).

Variables	Label	Fetal outcome		COR 95% CI	*p* value
		Died	Alive		
Age	19 or below	4 (18.2%)	18 (81.8%)	1.2 (0.37,3.90)	0.76
	20–24	10 (12%)	73 (88%)	0.74 (0.30,1.65)	0.46
	25–29	22 (15.6%)	119 (84.4%)	1	
	30–34	10 (14.9%)	57 (85.1)	0.95 (0.42,2.14)	0.90
	35 or above	16 (22.5%)	55 (77.5%)	1.57 (0.77,3.23)	0.22^*∗*^
Residence	Urban	2 (4%)	48 (96%)	0.19 (0.04,0.81)	**0.02**
	Rural	60 (18%)	274 (82%)	1	
Parity of women	Primipara	19 (17.4%)	90 (82.6%)	1.36 (0.68,2.71)	0.38
	Multipara	19 (13.5%)	122 (86.5%)	1	
	Grand multipara	24 (17.9)	110 (82.1%)	1.40 (0.73,2.70)	0.31
Frequency of ANC follow-up	<4	30 (20.7%)	115 (79.3%)	1.37 (0.75,2.50)	0.30
	≥4	23 (16%)	121 (84%)	1	
	Not mentioned	9 (9.5%)	86 (90.5%)	0.55 (0.23,1.25)	0.15^*∗*^
Women remembered LNMP	Yes	13 (16.9%)	64 (83.1%)	1.1 (0.55,2.1)	0.84
	No	49 (16%)	258 (84%)	1	
Cervical dilatation	Fully dilated	28 (23.7%)	90 (76.3%)	1	
	Not Fully dilated	34 (12.8%)	232 (87.2%)	0.47 (0.27,0.82)	0.01^*∗*^
Condition of membrane	Intact	9 (5.8%)	146 (94.2%)	1	
	Ruptured	53 (23.1%)	176 (76.9%)	4.89 (2.33,10.23)	**<0.001** ^*∗*^
Time duration of membrane rupture	<8 hours	37 (20.3%)	145 (79.7%)	1	
	≥8 hours	15 (31.9%)	32 (68.1%)	1.84 (0.90,3.74)	**0.09** ^*∗*^
Neonatal birth weight	<2500 gm	23 (43.4%)	30 (56.6%)	5.51 (2.85,10.63)	**<0.001** ^*∗*^
	2500–3500 gm	32 (12.2%)	230 (87.8%)	1	
	>3500 gm	7 (10.1%)	62 (89.9%)	0.81 (0.34,1.93)	0.64

Mode of delivery	Cs	8 (11.9%)	59 (88.1%)	0.66 (0.3,1.46)	0.31
	Vaginal delivery	54 (17%)	263 (83%)	1	
Gestational age by fundal height	<37	14 (19.7%)	57 (80.3%)	0.86 (0.16,4.6)	0.86
	37–42	33 (14.2%)	200 (85.8%)	0.6 (0.12,2.9)	0.51
	>42	2 (22.2%)	7 (77.8%)	1	

^*∗*^Significant at *p* < 0.25 (candidate variables for multivariable logistic regression model).

**Table 2:** Factors associated with perinatal outcome of breech delivery in Wolisso Hospital, 2013–2015.

Variables	Label	Fetal outcome		COR 95% CI	AOR 95% CI	*p* value
		Died	alive			
Age	19 or below	4 (18.2%)	18 (81.8%)	1.2 (0.37,3.90)	2.66 (0.71,9.94)	0.15
	20–24	10 (12%)	73 (88%)	0.74 (0.30,1.65)	0.58 (0.23,1.43)	0.24
	25–29	22 (15.6%)	119 (84.4%)	1	1	
	30–34	10 (14.9%)	57 (85.1)	0.95 (0.42,2.14)	1.45 (0.59,3.60)	0.42
	35 or above	16 (22.5%)	55 (77.5%)	1.57 (0.77,3.23)	2.62 (1.14,6.03)	**0.02**

Residence	Urban	2 (4%)	48 (96%)	0.19 (0.04,0.81)	0.22 (0.05,1.02)	0.052
	Rural	60 (18%)	274 (82%)	1	1	

Cervical dilatation	Fully dilated	28 (23.7%)	90 (76.3%)	1	1	
	Not Fully dilated	34 (12.8%)	232 (87.2%)	0.47 (0.27,0.82)	0.48 (0.25,0.91)	**0.024**

Condition of membrane	Intact	9 (5.8%)	146 (94.2%)	1	1	
	Ruptured	53 (23.1%)	176 (76.9%)	4.89 (2.33,10.23)	5.11 (2.25,11.60)	**<0.001**

Neonatal birth weight	<2500 gm	23 (43.4%)	30 (56.6%)	5.51 (2.85,10.63)	6.77 (3.22,14.25)	**<0.001**
	2500–3500 gm	32 (12.2%)	230 (87.8%)	1	1	
	>3500 gm	7 (10.1%)	62 (89.9%)	0.81 (0.34,1.93)	0.78 (0.31,1.96)	0.598

## Abbreviations

ANC: Antenatal care
OR: Odds ratio
RR: Relative risk
CI: Confidence interval
ECV: External cephalic version
MCH: Maternal and child health
St.: Saint
PROM: Premature rupture of membrane
SPSS: Statistical Package for Social Science
CNS: Central nervous system
C/s: Caesarean section
IUDF: Intrauterine fetal death
SGA: Small for gestational age
NICU: Neonatal intensive care unit.

## Additional Points

The data was collected by review of the registration books using structured checklists.
