# Corrigendum to “Prevalence and Perinatal Outcomes of Singleton Term Breech Delivery in Wolisso Hospital, Oromia Region, Southern Ethiopia: A Cross-Sectional Study”

**DOI:** 10.1155/2018/2143031

**Published:** 2018-02-26

**Authors:** Temesgen Debero Mere, Tilahun Beyene Handiso, Abera Beyamo Mekiso, Markos Selamu Jifar, Shabeza Aliye Ibrahim, Degefe Tadele Belato

**Affiliations:** ^1^Homecho Hospital, SNNPRS, Homecho, Ethiopia; ^2^Institute of Health, Jimma University, Department of Epidemiology, Jimma, Ethiopia; ^3^Department of Public Health, Wolaita Sodo University, Sodo, Ethiopia; ^4^Misha District Health Office, SNNPRS, Morsito, Ethiopia; ^5^Department of Statistics, Wachemo University, Hossana, Ethiopia

In the article titled “Prevalence and Perinatal Outcomes of Singleton Term Breech Delivery in Wolisso Hospital, Oromia Region, Southern Ethiopia: A Cross-Sectional Study” [1], the name of the sixth author was given incorrectly as Bilato. The author's name should have been written as Belato. The revised authors' list is shown above.
